# Regional Control of *Drosophila* Gut Stem Cell Proliferation: EGF Establishes GSSC Proliferative Set Point & Controls Emergence from Quiescence

**DOI:** 10.1371/journal.pone.0080608

**Published:** 2013-11-13

**Authors:** Marie Strand, Craig A. Micchelli

**Affiliations:** Department of Developmental Biology, Washington University School of Medicine, St. Louis, Missouri, United States of America; University of Bern, Switzerland

## Abstract

Adult stem cells vary widely in their rates of proliferation. Some stem cells are constitutively active, while others divide only in response to injury. The mechanism controlling this differential proliferative set point is not well understood. The anterior-posterior (A/P) axis of the adult *Drosophila* midgut has a segmental organization, displaying physiological compartmentalization and region-specific epithelia. These distinct midgut regions are maintained by defined stem cell populations with unique division schedules, providing an excellent experimental model with which to investigate this question. Here, we focus on the quiescent gastric stem cells (GSSCs) of the acidic copper cell region (CCR), which exhibit the greatest period of latency between divisions of all characterized gut stem cells, to define the molecular basis of differential stem cell activity. Our molecular genetic analysis demonstrates that the mitogenic EGF signaling pathway is a limiting factor controlling GSSC proliferation. We find that under baseline conditions, when GSSCs are largely quiescent, the lowest levels of EGF ligands in the midgut are found in the CCR. However, acute epithelial injury by enteric pathogens leads to an increase in EGF ligand expression in the CCR and rapid expansion of the GSSC lineage. Thus, the unique proliferative set points for gut stem cells residing in physiologically distinct compartments are governed by regional control of niche signals along the A/P axis.

## Introduction

The decision of whether or not a cell should divide is fundamental. For adult stem cells this choice is of particular importance, since stem cells not only replace differentiated cells during normal tissue turnover, but are also capable of massive lineage expansion following injury or transformation. Advances in cell lineage tracing methodology have made it possible to precisely ascertain the location of resident stem cell populations *in vivo* [1]. In the most rapidly renewing tissues, such as the skin, blood, and gut, adult stem cells can be classified according to their relative rates of proliferation; some are constitutively active while others are quiescent and only activated in response to injury [2]. A complex interplay of niche factors is necessary to orchestrate stem cell behavior in these actively renewing tissues. Yet, precisely how a core niche program is regulated to selectively control the behavior of distinct stem cell populations remains poorly understood. 

The *Drosophila* midgut has proven to be of great value in the study of adult tissue homeostasis [3]. This organ system is lined with an epithelial monolayer that exhibits a segmental organization along the anterior-posterior (A/P) axis. Grossly, the adult gastrointestinal epithelium presents few anatomical landmarks to reliably determine cellular position along its length [4]. However, a number of distinct cell types have been classically recognized based on their morphology or ability to concentrate dietary nutrients, such as the copper cells, large flat cells and iron cells of the middle midgut [4-6]. Attempts to standardize midgut regionality were originally based on dividing the tissue into domains of equivalent length in both the anterior (i.e. A1-4) and posterior (i.e. P1-4) midgut [7]. Panels of molecular markers were subsequently employed to generate higher resolution maps that subdivided the epithelium into discrete domains based on gene expression [8]. Recent studies have employed genomic approaches to build upon existing maps of the adult gut epithelium [9,10]. These studies have added most significantly to our understanding by establishing useful molecular landmarks in the otherwise featureless anterior and posterior regions of the gut. Thus, the adult midgut epithelium is divided into a series of segmental regions that can be indexed by a combination of morphology, function and gene expression.

The differentiated gut epithelial cell types that typify each region of the midgut are maintained by identified stem cells. Gut stem cells can be distinguished on the basis of their position along the gastrointestinal (GI) tract, molecular markers, cell lineage and proliferative rate [8,10-12]. For example, gastric stem cells (GSSCs) reside in the highly acidic copper cell region of the middle midgut, while intestinal stem cells (ISCs) of the posterior midgut reside in a neutral compartment specialized for nutrient absorption. Gastric stem cells are further distinguished from ISCs by their ability to generate acid-secreting cells of the CCR and by their relative quiescence [8,10]. GSSCs normally divide at very low rates, but can also be stimulated to rapidly regenerate the gastric epithelium following acute injury by enteric pathogens or heat stress [8]. And yet, the molecular mechanism underlying regional differences in adult gut stem cell proliferation have not been investigated. In this study, we demonstrate that the conserved epidermal growth factor (EGF) signaling pathway is differentially regulated in the adult *Drosophila* copper cell region and is a key factor controlling the episodic emergence of GSSCs from their quiescent state.

## Results

### Gastric epithelium of the adult middle midgut

**Figure 1:**
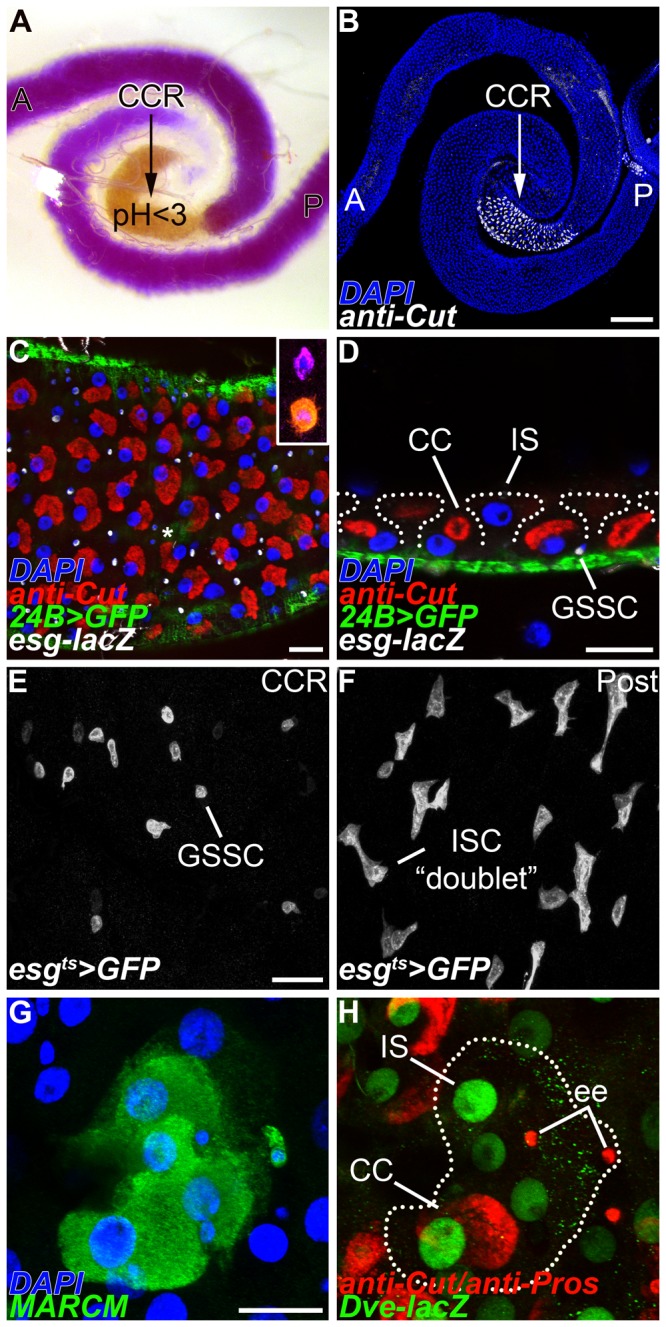
Tripotent gastric stem cells maintain the copper cell region. (A-B) The adult copper cell region (CCR) is a physiologically distinct region of the midgut; anterior (A), posterior (P). (A) Feeding with the pH indicator dye bromophenol blue reveals the acidic copper cell region with a pH <3 (yellow). (B) Anti-Cut specifically marks the acid-secreting copper cells that give rise to the acidic compartment present in A. (C-D) *esg-lacZ* expressing gastric stem cells (GSSC, white) are interspersed among copper cells (CC, anti-Cut^+^, red) and are located basally in the epithelial layer in close proximity to the surrounding visceral muscle (24B>*GFP* ^+^, green). (C) Superficial section. Asterisk shows enteroendocrine cells. Inset shows a pair of enteroendocrine cells that can be further characterized by neuropeptide expression, Allatostatin C (purple) and Neuropepide F (orange). (D) Cross-section. (E-F) *esg*>*GFP* marks progenitor cells in the adult midgut. (E) Quiescent *esg*>*GFP* ^+^ gastric stem cells (GSSC) in the copper cell region (CCR) are typically found as single small rounded cells. (F) Actively dividing *esg>GFP* ^+^ intestinal stem cells and their undifferentiated daughters are typically present as “doublets” in the posterior midgut (Post). (G-H) MARCM labels a tripotent gastric stem cell lineage in the CCR. (G) A GFP labeled MARCM clone (anti-GFP^+^, green). (H) The same clone shown in G spans all three differentiated cells types found in the region; copper cells (CC, anti-Cut^+^, red), interstitial cells (IS, *Dve-lacZ* ^*+*^, green), and enteroendocrine cells (ee, anti-Pros^+^, red). Scale bars: 200μm in B, 20μm in C, D, E and G.

The *Drosophila* copper cell region (CCR) or “stomach”, is a highly acidic region with a pH<3 that is flanked by the more alkaline regions of the anterior and posterior midgut [13,14]. This distinct physiology is readily evident when colorimetric pH indicator dyes, such as bromophenol blue, are fed to adult flies (Figure 1A). Acidification of the middle midgut is the result of acid-secreting copper cells [13]. Copper cells were originally named for their ability to concentrate dietary copper, which can be detected by emission of strong orange fluorescence [5,15]. However, labeling acid-secreting cells with dietary copper (CuCl_2_) can be both variable and labile. A panel of molecular markers has been used to generate a detailed cellular map of the gastric epithelium and includes Cut, which reproducibly marks adult copper cells (Figures 1B and S1) [8]. While the entire extent of the middle midgut epithelium is molecularly defined by the transcription factor *defective proventriculus* (*dve*), acid-secreting copper cells comprise only a regionally delimited subdomain of epithelial cells (Figures S1B and S1C) [8]. In addition, the copper cell region contains two other cell types of poorly characterized function: interstitial cells and enteroendocrine cells. Interstitial cells are interspersed between copper cells and have more apically positioned nuclei (Figures 1D and S1D). Enteroendocrine cells in the CCR are of two subtypes that can be distinguished by the expression of unique secretory neuropeptides (Figures 1C inset, and S2) [16]. Dispersed among the differentiated cells of the CCR are *esg* ^+^ gastric stem cells (GSSCs), which are reproducibly positioned in close apposition to the surrounding visceral muscle (Figures 1C, 1D and S1A). Consistent with arrest in a quiescent state [8,10], *esg* ^+^ cells in the CCR region are present as individual gastric stem cells (Figures 1C and 1E) and not the “doublets” typically observed in the rapidly proliferating intestinal stem cells of the posterior midgut (Figure 1F) [17]. 

### Tripotent gastric stem cells maintain the CCR epithelium

An unresolved issue in the CCR is the precise cell lineage of individual GSSCs. Our previous analysis of wild type cell lineages showed that GSSCs are multipotent [8]. We wished to extend this initial mosaic analysis to more rigorously define the distribution of gastric epithelial cell fates in marked clones. We used the MARCM system to label dividing cells with GFP [18]. Transient clones lacking GSSCs generated during the labeling pulse are completely purged from the epithelium 10 days later, leaving only expanded gastric stem cell lineages [8]. Therefore, we combined MARCM labeling with a panel of molecular and morphological markers for copper, interstitial, and enteroendocrine cells, to classify GSSC lineages 14 days after induction (Table 1). These experiments showed that wild type GSSC lineages in the CCR contained all three differentiated cell types in 26% of the cases analyzed (n=78 clones) (Figures 1G and 1H). 35% of the clones contained two differentiated cell types, usually a mixture of copper and interstitial cells; 24% of the clones contained only one differentiated cell type; while the remaining 15% of clones did not stain for markers of differentiated cells and contained only progenitor cells. All of the clones with differentiated cells contained interstitial and/or copper cells, whereas only 33% contained enteroendocrine cells, suggesting that enteroendocrine cells are made less frequently, or later in temporal order than copper and interstitial cells. Thus, GSSCs are minimally tripotent and give rise to the acid-secreting copper cells, absorptive interstitial cells, and secretory enteroendocrine cells found in the adult CCR.

**Table 1:** Wild type GSSC lineages analyzed 14 days after induction.

		**Number of differentiated cell types per GSSC clone**			
	**Total**	**3**	**2**	**1**	**0**
Number	78	20	27	19	12
Percent	100%	26%	35%	24%	15%

### Reporters of EGF signaling are induced in the CCR following infection

**Figure 2:**
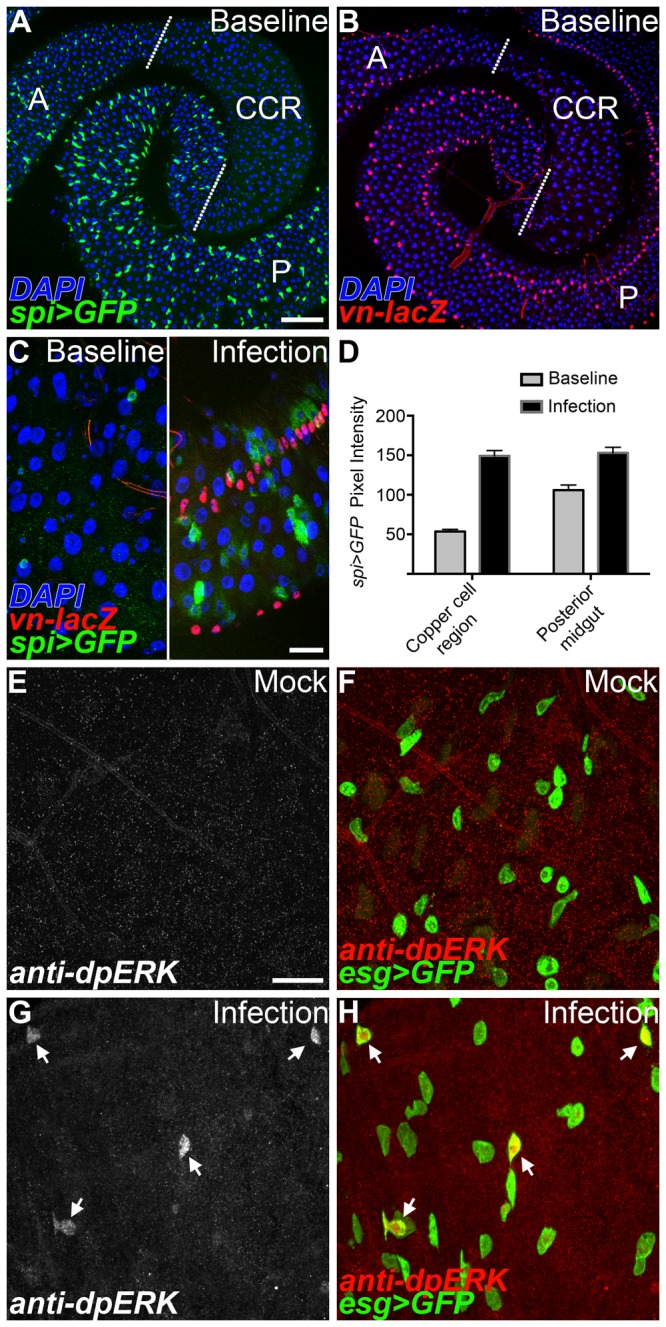
EGF signaling is low in the copper cell region but strongly induced by infection. (A-C) Under baseline conditions EGF ligand reporters are highly expressed in the anterior (A) and posterior (P) midgut regions, whereas expression is low in the copper cell region (CCR). (A) Expression of the *spi*>*GFP* reporter. (B) Expression of the *vn-lacZ* reporter. (C) High magnification view of the copper cell region. EGF ligand reporters are induced in the copper cell region 24 hrs after enteric infection (right panel). (D) Quantification of *spi*>*GFP* ^+^ cells pixel intensities in both the copper cell region and posterior midgut under baseline and infected conditions. A significant increase in reporter levels is observed in both the CCR and posterior midgut following *Pe* infection (n=60 cells/condition, p<0.0001). (E-H) Anti-dpERK (white/red) staining is low in gastric stem cells (*esg*>*GFP* ^+^, green) in the copper cell region but induced 4 hrs following bacterial infection. (E-F) Anti-dpERK staining (white/red) is low/absent in mock infected controls. (G-H) Infection induced anti-dpERK staining (arrows) colocalizes with gastric stem cells (*esg*>*GFP* ^+^, green). Scale bars: 100μm in A, 20μm in C and E.

Cell lineage tracing analysis has shown that GSSCs are the most quiescent stem cell population in the adult gut [8,10]. GSSCs can be induced to quickly emerge from quiescence and regenerate the gastric epithelium in response to environmental challenge, such as high titer exposure to the Gram-negative pathogen *Pseudomonas entomophila* (*Pe*) or heat stress [8]. We hypothesized that the EGF signaling pathway might control gastric stem cell proliferation in the CCR, as EGF is known to regulate the actively proliferating intestinal stem cells in the posterior midgut [19-22]. Flies contain a single EGF receptor (EGFR) that binds to one of four activating ligands: Gurken (Grk), Keren (Krn), Spitz (Spi), and Vein (Vn) [23].  Ligand binding leads to receptor phosphorylation, activation of the Ras/MAPK cascade, and phosphorylation of the extracellular signal-regulated kinase (Erk). To measure EGF/MAPK pathway activity in the CCR, we analyzed reporters of ligand expression and stained for the presence of phosphorylated Erk protein. All measures of EGF activity analyzed consistently showed that the EGF pathway is normally inactive in the CCR. For example, expression of the *spi*>*GFP* and *vn-lacZ* reporters were both low under baseline conditions in the CCR (Figures 2A-2C).  77% of adult midguts had little to no detectable *spi*>*GFP* reporter expression in the CCR under baseline conditions (n=62). This observation was in marked contrast to the high expression of these reporters seen in both the anterior and posterior regions of the midgut (Figures 2A and 2B) [20,22].  We then quantified the level of EGF ligands present in the midgut (Figures 2D and S3E). These measurements show that at baseline, significantly lower levels of EGF ligands were present in the CCR compared to the posterior midgut. Gurken was not detected anywhere in the midgut under baseline conditions (Figure S3A). Therefore, in the CCR where GSSCs are quiescent, EGF ligands were either low or undetectable at baseline.

We next examined the expression of EGF reporter lines following acute exposure to *Pe*, a challenge which activates GSSC proliferation [8].  In contrast to uninfected controls, EGF ligands were rapidly and robustly induced in the adult CCR following enteric infection (Figures 2C, 2D and S3C-E). For example, 81% of samples analyzed displayed high levels of *spi*>*GFP* reporter expression in the CCR (n=16). Quantification showed that following infection, *spi*>*GFP* expression was induced to the same level in both the CCR and posterior midgut, despite initial differences under baseline conditions (Figures 2D). Finally, molecular markers of the gut epithelium and the surrounding visceral muscle were then used to more finely localize induced EGF ligand expression after challenge. Double labeling showed that *spi*>*GFP* is induced in *esg-lacZ* ^*+*^ progenitor cells in the gastric epithelium, while *vn-lacZ* expression is induced in 24B>*GPF* ^+^ cells of the surrounding visceral muscle (Figures S3C and S3D). No detectable changes in Gurken expression were observed in the CCR following challenge (Figure S3B).   

To confirm EGF reporter analysis, we next examined EGF pathway activation using an antibody raised against the diphosphorylated form of the Erk kinase (dpERK). Consistent with ligand expression, anti-dpERK staining is rarely observed in the adult CCR under baseline conditions (Figures 2E and 2F). If any staining was detected, the signal was weak and found only in a small fraction of gastric stem cells. In contrast, challenge with *Pe* led to a striking increase in anti-dpERK staining in *esg* ^+^ cells (Figures 2G and 2H). The anti-dpERK staining observed following infection was comparable to the levels observed when the EGF pathway was autonomously activated in the gastric stem cell population (Figure S3F). Taken together, analysis of EGF signaling shows that pathway activity is low in the CCR under baseline conditions when GSSCs are quiescent, but high after *Pe* challenge when GSSCs are induced to divide. These findings suggest that EGF is transduced in GSSCs and therefore, may have a direct functional role in controlling the emergence of GSSCs from quiescence.

### EGF activation is sufficient to induce GSSC proliferation

**Figure 3:**
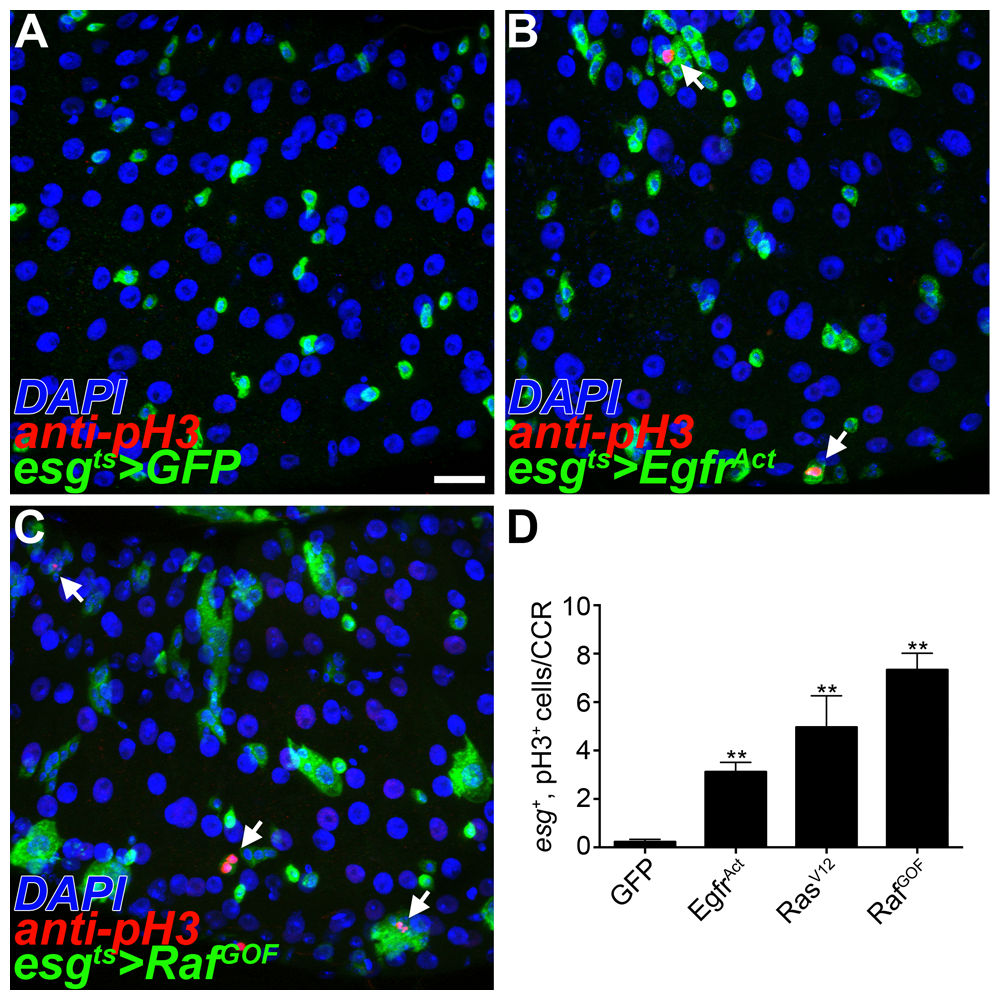
EGF signaling is sufficient to induce gastric stem cell proliferation. (A-D) Conditional activation of EGF signaling under control of the *esg* promoter in the copper cell region (CCR); *esgGal4,UAS-GFP,tubGal80* ^*ts*^ (*esg* ^*ts*^, anti-GFP^+^, green); dividing cells (anti-pH3^+^, red). (A) In control experiments few pH3^+^ cells are observed. (B-C) Activation of EGF signaling in gastric progenitor cells leads to an increased number of pH3^+^ cells (arrows). (B) Constitutively active form of the EGF receptor (C) Constitutively active form of the Raf kinase. (D) Quantification of *esg* ^+^, pH3^+^ cells in the CCR (n=24-26 guts/genotype, error bars ±SEM, ** p<0.0005). Scale bar: 20μm.

To test the sufficiency of EGF to induce GSSC proliferation, we conditionally expressed downstream components of the pathway in the absence of environmental challenge. We used the *Gal4/UAS* system in combination with a temperature sensitive *Gal80* repressor to conditionally drive transgene expression in the *esg* ^+^ cells of the gastric epithelium (*esgGal4,UAS-GFP,tubGal80* ^*ts*^; hereafter *esg* ^*ts*^). Flies were reared at the permissive temperature to ensure normal development and adults were then shifted to the restrictive temperature. GSSC proliferation was assayed using anti-phosphohistone H3 (pH3) to label dividing cells. In control guts expressing *GFP* alone, little or no pH3 staining was detected in the adult CCR, again consistent with the idea that GSSCs are normally quiescent (Figures 3A, 3D, S4A and S4B). However, ectopic EGF signaling significantly increased the number of *esg* ^+^, pH3^+^ cells in the CCR in a rapid and sustained manner (Figures 3B-3D, S4A and S4B). Thus, EGF signaling is sufficient to activate quiescent GSSCs in the absence of environmental challenge. 

We further tested the sufficiency of EGF signaling to induce GSSC proliferation using MARCM. Components of the EGF signaling pathway were expressed mosaically in GSSC lineages and analyzed two weeks after induction to determine the number of cells per clone for each genotype (Figures S4C and S4D). The distribution of GSSC clone sizes was altered by activating EGF using either the *Egfr* ^*Act*^ or *Raf* ^*GOF*^ construct. Consistent with the increased pH3 staining observed following conditional activation of EGF in GSSCs (Figure 3), large clones (>20 cells) were more frequently recovered in GSSC lineages expressing *Raf* ^*GOF*^ than in wild type controls (Figure S4D). However, this effect was not reflected in the average number of cells per clone, as mosaic activation of EGF also led to the recovery of many small clones (Figure S4C and S4D). This is likely due to additional roles for EGF signaling in the GSSC lineage [19]. Therefore, EGF pathway activation is sufficient to stimulate proliferation and in some cases to drive clonal expansion of GSSC lineages even in the absence of challenge.

### EGF signaling is required for GSSC proliferation

**Figure 4:**
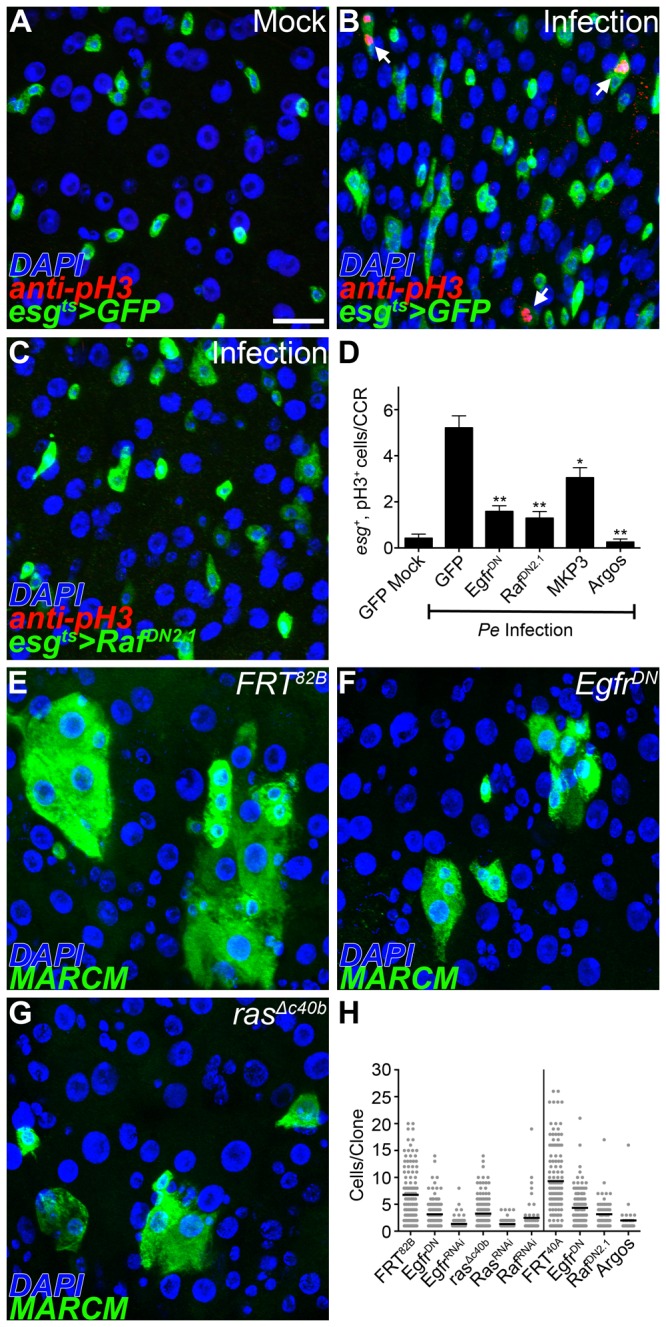
EGF signaling is necessary for gastric stem cell proliferation. (A-D) Conditional loss of EGF signaling under control of the *esg* promoter in the copper cell region (CCR); *esgGal4,UAS-GFP,tubGal80* ^*ts*^ (*esg* ^*ts*^, anti-GFP^+^, green); dividing cells (anti-pH3^+^, red). Flies were subjected to a 16 hr enteric infection to induce gastric stem cell proliferation. (A) In mock-infected controls few pH3^+^ cells are observed. (B) Enteric infection leads to an increased number of pH3^+^ cells (arrows). (C) A dominant-negative form of the Raf kinase abrogates pH3^+^ levels following infection. (D) Quantification of *esg* ^+^, pH3^+^ in the CCR 16 hrs after enteric infection (n=23-24 guts/genotype, error bars ±SEM, * p<0.005, ** p<0.0005). (E-H) MARCM labeling of gastric stem cell lineages in the copper cell region 14 days after induction (anti-GFP^+^, green). (E) Wild type gastric stem cell lineages. (F) Gastric stem cell lineages expressing a dominant-negative form of the EGF receptor. (G) Gastric stem cell lineages completely lacking *ras*. (H) Quantification of the number of cells per clone for each genotype analyzed (n=35-163 clones/genotype). All EGF loss-of-function clones examined show significantly fewer cells/clone than controls (p<0.0005). Scale bar: 20μm.

We next tested the necessity of the EGF pathway for GSSC proliferation in response to challenge. The conditional *esg* ^*ts*^ driver line was first used to block EGF signaling and flies were then subjected to *Pe* infection. The number of pH3^+^ cells in the adult CCR was scored 16 hours following infection. In mock-infected controls, very few pH3^+^ cells were detected (Figures 4A and 4D). When control flies expressing *GFP* alone were challenged with *Pe* the number of pH3^+^ cells in the CCR increased significantly (Figures 4B and 4D). We also noted an associated increase in *esg* ^+^ cell number, another indication of actively dividing cells. However, these effects were abrogated when EGF signaling was blocked in the *esg* ^+^ cell population (Figures 4C and 4D). Similar phenotypes were observed using inactivating constructs for different components of the pathway, including dominant-negative forms of *Egfr* or *Raf* and by ectopically expressing negative regulators of the pathway such as MAP kinase phosphatase 3 (MKP3) or *argos*. Thus, functional EGF signaling is necessary in the *esg* ^+^ population for GSSCs to emerge from their quiescent state following challenge. 

We extended this analysis by testing the requirement of EGF signaling in GSSC lineages. Again, using clonal analysis we scored the number of cells per clone as a measure of GSSC proliferation. Wild type control clones had, on average, 6-9 cells per clone (Figures 4E and 4H; *FRT* ^82B^ *, FRT* ^40A^) however a significant reduction was observed when EGF signaling was blocked (Figures 4F-4H). Complete loss of EGF signaling, in *ras* null clones, also resulted in fewer cells per clone than controls (Figures 4G and 4H). Some GSSCs were still able to generate daughter cells even when EGF signaling was completely eliminated. We determined the identity of these daughter cells in EGF loss-of-function clones using established molecular markers of the gastric epithelium. Prospero^+^ enteroendocrine cells and Dve^+^ polyploid cells, marking both copper and interstitial cells, were recovered in mutant GSSC lineages (Figure S5). Taken together, these experiments demonstrate that EGF signaling is required cell-autonomously for GSSCs to emerge from quiescence, but is dispensable for subsequent lineage differentiation. 

## Discussion

**Figure 5:**
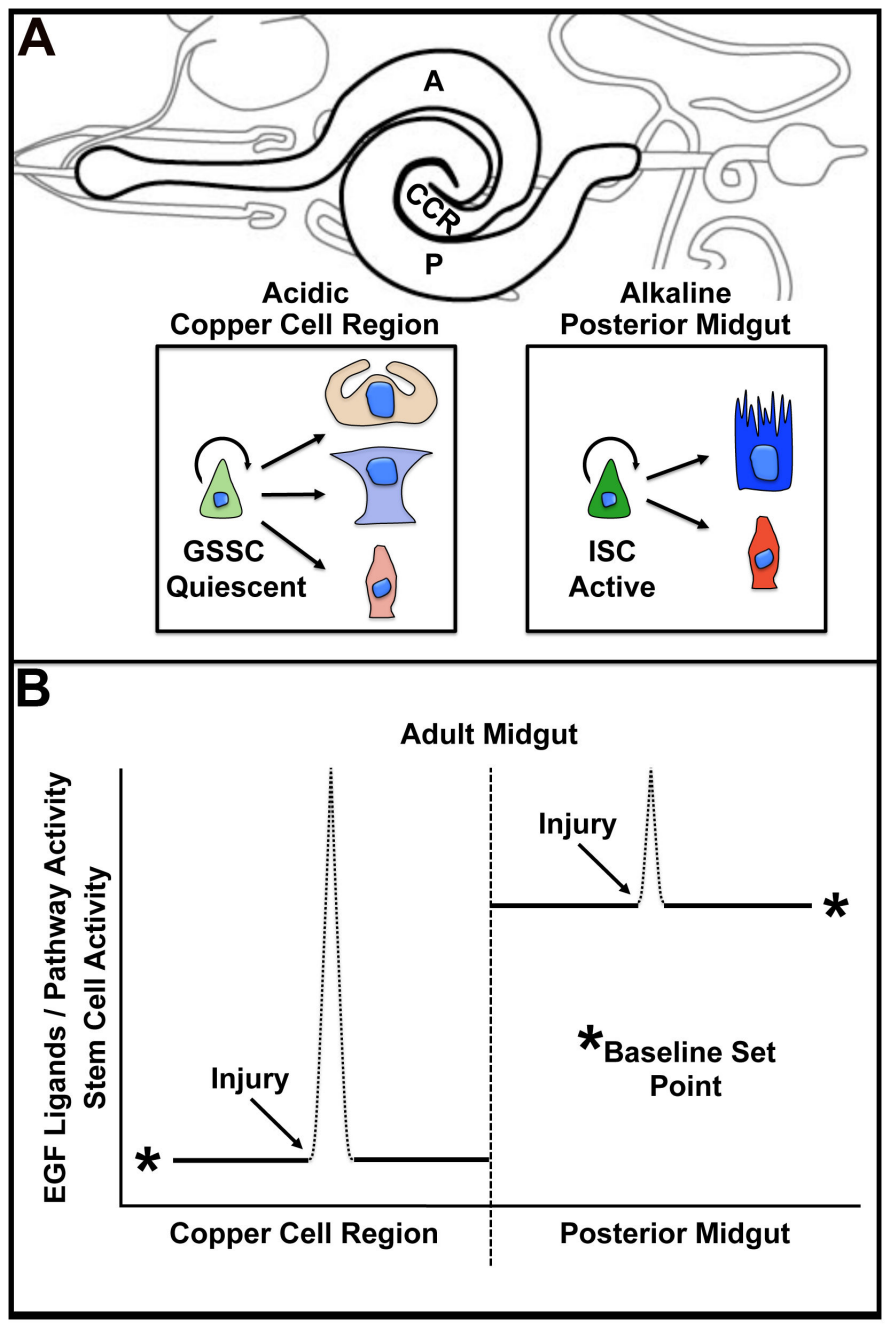
Regional control of gut stem cell proliferation. (A) Schematic diagram of the adult *Drosophila* midgut (bold). The gut can be divided into physiologically distinct regions; anterior (A), copper cell (CCR) and posterior (P). Gastric stem cells (GSSCs) in the acidic CCR differ from intestinal stem cells (ISCs) in the alkaline posterior midgut in two ways. First, GSSCs are tripotent and maintain three unique cells types in the lineage: acid-secreting copper cells (orange), interstitial cells (blue), and enteroendocrine cells (red). ISCs are bipotent and produce enterocytes (dark blue) and enteroendocrine cells (dark red). Second, GSSCs are quiescent compared to the more actively proliferating ISCs. (B) A model explaining the regional difference in gut stem cell proliferation. Gastric stem cells of the CCR are controlled by region specific modification of a core niche program. Under baseline conditions, EGF signaling and stem cell activity are low in the copper cell region and high in the posterior midgut. Injury induces EGF ligands and activates stem cells to divide.

The CCR epithelium is the exclusive site of large acid-secreting copper cells responsible for generating a low pH compartment in the midgut. Gastric stem cells in the CCR are normally quiescent but are robustly stimulated to replenish the unique differentiated cells of the gastric epithelium in response to injury by enteric pathogens or heat stress. In this study, we resolve outstanding issues related to the GSSC lineage, demonstrating the presence of tripotent GSSC lineages in the CCR. In addition, we demonstrate a central role for the conserved EGF signaling pathway in controlling the emergence of gastric stem cells from quiescence. Taken together, two key differences between GSSCs and ISCs are now evident (Figure 5A): the unique region specific cell lineages that they support (copper, interstitial, enteroendocrine vs. enterocyte and enteroendocrine) and their activity levels (quiescent vs. active). Thus, maintenance of physiologically and functionally distinct compartments of the adult midgut depends upon the activity of distinct stem cell lineages.

What is the nature of the unique molecular program that governs the observed differences in GSSC and ISC proliferative behavior? Our study indicates that regional differences in gut stem cell proliferation are controlled by regional differences in EGF ligand availability (Figure 5B). First, reporters of EGF pathway activity are normally very low in the CCR under baseline conditions, when GSSCs are quiescent. However, damage to the gastric epithelium by enteric infection increases local EGF ligand expression and Erk phosphorylation. This EGF activation directly correlates with an observed increase in proliferating GSSCs. Second, ectopic activation of the EGF pathway is sufficient to cell-autonomously promote GSSC proliferation in the absence of environmental challenge. Finally, functional EGF signaling is required for GSSC proliferation following enteric infection and for GSSC lineage expansion. Importantly, our studies of GSSCs in the CCR are similar to previous studies demonstrating that EGF signals are an essential part of the core niche program controlling the ISC lineage [19-22]. Thus, regional control of EGF ligands, and perhaps other regulators of EGF pathway activity, are essential in generating gastrointestinal stem cell niches with distinct proliferative set points. 

In this light, it is worth noting that over-expression of epidermal growth factors and their receptors are associated with human gastric cancer, the second leading cause of cancer-related deaths worldwide [24,25]. In addition, Ménétrier’s disease is a hyperproliferative disorder of the stomach caused by over-expression of the EGF ligand TGF-α. Over production of TGF-α and increased EGF signaling is associated with an expansion of surface mucous cells and a reduction in parietal and chief cells [26]. Gastric stem cells are the proposed cell-of-origin in Ménétrier’s disease, but this has not been directly tested due to a lack of gastric stem cell specific markers in the murine system. Advances in understanding how EGF ligand availability controls activity of the acid-secreting gastric stem cell lineage in *Drosophila* raises the possibility that hyperplastic conditions associated with the human stomach might arise when ectopic EGF ligands draw resident stem cells out of their quiescent state.

EGF signaling appears to be only one aspect of the region specific program controlling gastric stem cells in the adult copper cell region. We previously reported that a *Delta-lacZ* enhancer trap line was not present in GSSCs under baseline conditions [8]. In the course of this study, we observed that *Pe* challenge also leads to an increase in *Delta* ligand expression in dividing cells (Figure S6), suggesting a role for Delta/Notch signaling in the GSSC lineage. In addition, elegant studies of GSSCs under baseline conditions have recently shown that the secreted BMP/Dpp signaling pathway is both necessary and sufficient to specify copper cells in the adult midgut and acts via the *labial* transcription factor [9,27,28]. Interestingly, while the highest levels of Dpp pathway reporters are detected in the CCR, manipulation of the BMP/Dpp pathway did not affect GSSC proliferation [27,28]. Thus, the GSSC lineage is influenced by secreted niche factors, which independently control both GSSC proliferation and cell fate specification. 

In conclusion, understanding GI regionality and homeostatic diversity along the A/P axis is important for several reasons. First, as highlighted by the work presented here, we can gain insight into how the modification of a core GI niche program, which adapts each stem cell to its compartment specific physiology, leads to difference in lineage output. Second, disruption of regional identity along the GI tract is associated with a class of precancerous conditions called metaplasias, in which one region of the GI tract takes on the attributes of another [29]. Finally, both the establishment and maintenance of tumorigenic lineages exhibit marked preferences along the A/P axis of the gut [25]. The striking similarities between vertebrate and invertebrate GI biology, suggest that delving deeper into the mechanisms underlying *Drosophila* midgut regionalization will continue to provide important insights into these fundamental biological problems. 

## Materials and Methods

### Fly stocks

w^1118^; w;esg^Gal4^,UAS-GFP/CyO; w;esg^Gal4^,UAS-GFP,tubGal80^ts^ (esg^ts^); w;sp/CyO;24B^Gal4^,UAS-GFP; cn^1^,dve-lacZ^100738^/CyO;ry^506^; w;spi^Gal4^/CyO;UAS-mCD8-GFP/TM6C; w;esg^K606^/CyO; w;NP2788^Gal4^,UAS-GFP/CyO;tubGal80^ts^/TM6C; w;NPF^Gal4^/CyO; y,w;+;UAS-mCD8-GFP; cn^1^;vn-lacZ^10567^,ry^506^/TM3; y,w;vn-lacZ^P1719^,FRT^80B^; y,w;UAS-Egfr^DN^;UAS-Egfr^DN^; w;sp/CyO;UAS-Raf^DN2.1^; w;UAS-Argos^232^; y,w;sp/CyO;UAS-MKP3/TM6B; w;+;UAS-TorD-DER (UAS-Egfr^Act^); w;UAS-Ras^V12^/CyO;Dr/TM6C; w;+;UAS-Raf^GOF^; w;UAS-Egfr^RNAi^; w;UAS-Ras^RNAi^; w;UAS-Raf^RNAi^; y,w,UAS-GFP,hsflp;FRT^40A^,tubGal80;tubGal4/TM6B; w;FRT^40A^,hsπM;+; y,w,UAS-GFP,hsflp;+;tubGal4,FRT^82B^,tubGal80/TM6B; w;+;FRT^82B^,hsπM; Dl^Gal4^; Su(H)GBE^Gal4^.

### Antisera

#### Primary antibodies

The following primary antibodies were used: chicken anti-GFP (1:10,000, Abcam); rabbit anti-βGal (1:2,000, Cappel); rabbit anti-pH3 (1:2,000, Upstate); mouse anti-βGal (1:100, Developmental Studies Hybridoma Bank); mouse anti-Pros (1:100, Developmental Studies Hybridoma Bank); mouse anti-Cut (1:100, Developmental Studies Hybridoma Bank); rabbit anti-Dve (1:2,000, generous gift from Dr. Fumio Matsuzaki ); rabbit anti-AstC (1:500, generous gift from Drs. Paul Taghert and Jan Veensta); mouse anti-Grk (1:50, Developmental Studies Hybridoma Bank); mouse anti-dpERK (1:100, Sigma).

#### Secondary antibodies

The following secondary antibodies were used: goat anti-chicken Alexa Flour 488 (1:2,000, Molecular Probes); goat anti-mouse Alexa Flour 568 (1:2,000, Molecular Probes); goat anti-mouse Alexa Flour 647 (1:2,000, Molecular Probes); goat anti-rabbit Alexa Flour 568 (1:2,000, Molecular Probes); goat anti-rabbit Alexa Flour 647 (1:2,000, Molecular Probes).

#### Mounting media and dyes

Vectashield+ DAPI mounting media (Vector) was used.

### Histology

Adult female flies were dissected in 1x PBS (Sigma). The midgut was removed and fixed 0.5x PBS and 4% electron microscopy-grade formaldehyde (Polysciences) overnight. Samples were washed in 1x PBS, 0.1% Triton X-100 (PBST) for a minimum of 2 hours, and then incubated in primary antibodies overnight. Samples were then washed for 2 hours in PBST, incubated in secondary antibodies for 3 hours, and then washed again for 2 hours in PBST. The final wash was removed and mounting media containing DAPI (Vectashield) was added for at least 2 hours before mounting. All steps were completed at 4 °C. For dpERK antibody staining, adult females were dissected in ice cold PBS. Midguts were fixed in 8% electron microscopy-grade formaldehyde (Polysciences) for 30 minutes at room temperature. Samples were then dehydrated in a series of methanol washes and rehydrated in PBST before antibody staining.

### Microscopy and imaging

Samples were analyzed on a Leica DM5000 compound fluorescence microscope. Confocal images were collected with a Leica TCS SP5 confocal microscope. All images were processed for brightness and contrast in Photoshop CS (Adobe).

### Bromophenol blue feeding

Bromophenol blue (BPB) pH indicator dye (Sigma) was dissolved in food at 0.1%. Staining is yellow at pH<3 and blue at pH>4.6. Flies were placed on 0.1% BPB food for 5-24 hours prior to dissection. BPB flies were dissected in ice cold PBS one at a time and analyzed immediately to avoid dissipation of staining. 

### MARCM clonal analysis

Positively marked cell lineages were generated in female midguts using the MARCM system. Fly crosses were cultured on standard media supplemented with yeast paste at 25 °C. Newly eclosed females of the appropriate genotype were aged 3-6 days prior to clone induction. Clones were induced by placing vials in a 37 °C water bath for 30-40 minutes. Flies were heat-shocked 3 times within a 24 hour period. For gain- and loss-of-function EGF clonal analysis, 3 independent experimental replicates were performed, from which 12 guts/genotype were analyzed 14 days after clone induction.

### Infection

Flies were infected ad libitum with *Pseudomonas entomophila* (Pe). Infected flies were fed on food supplemented with 0.5mL of *Pe* at OD_20_ in 5% sucrose. Mock-infected flied were placed on food supplemented with 0.5mL 5% sucrose. Flies were maintained at 29 °C throughout the course of infection. 

### Quantification of EGF ligands

Pixel intensities for the *spi*>*GFP* and *vn-lacZ* reporter lines were quantified with Leica TCS SP5 confocal software. To calibrate the detection range for each EGF reporter analyzed, PMT gain was set such that expression was detectable in the copper cell region under baseline conditions. These settings were then used to acquire all images, thus permitting the comparison of pixel intensities between regions and across all samples. The scale of pixel intensities ranged from 0-255 (255=saturation). 6 guts for each reporter line were imaged under both baseline and infected conditions. 10 cells from the copper cell region and posterior midgut were quantified in each gut. 

### EGF pathway analysis

#### Activation of the EGF signaling pathway is sufficient to induce GSSC proliferation

The *esg* ^*ts*^ driver line was used to active EGF signaling in adult GSSCs. Crosses were established and cultured at 18 °C until adulthood to ensure proper development. F1 female progeny of the appropriate genotype were aged at 18 °C for 4 days and then shifted to the restrictive temperature (29 °C). Flies were dissected 3 days after temperature shift and scored for the number of *esg* ^+^, pH3^+^ cells in the copper cell region.

#### EGF signaling is required in gastric progenitor cells for the proliferative response in GSSCs following infection

The *esg* ^*ts*^ driver line was used to inhibit EGF signaling in adult GSSCs. Crosses were established and cultured at 18 °C until adulthood to ensure proper development. F1 female progeny of the appropriate genotype were aged 3-5 days at 18 °C and then shifted to 29 °C. 3 days after temperature shift, flies were infected with *Pe* at OD_20_. Flies were dissected 16 hours after infection (4 days after temperature shift) and scored for the number of *esg* ^+^, pH3^+^ cells in the copper cell region.

## Supporting Information

Figure S1 **Molecular markers define specific cell types in the adult *Drosophila* copper cell region.** (A) *esg*>*GFP* marks diploid progenitor cells throughout the entire length of the adult midgut. (B) Anti-Dve expression defines the middle midgut region and marks all polyploid differentiated cells in the region. (C) Anti-Cut marks the acid-secreting copper cells in a subdomain of the middle midgut. (D) *NP2788>GFP* is expressed in a subset of interstitial cells found in the middle midgut. Anterior is to the left in all panels. Scale bar: 200μm.
(TIF)

Figure S2 **Enteroendocrine cells in the copper cell region can be further characterized based on neuropeptide expression.** (A) The neuropeptide F (NPF) Gal4 driver (*NPF*>*GFP* ^+^, green) is expressed in the copper cell region and in the flanking anterior and posterior midgut regions. Anti-Cut staining (red) marks the copper cell region of the middle midgut. Anterior is to the left. (B) High magnification image of the copper cell region (copper cells are marked by anti-Cut, red). The NPF diver is expressed in diploid cells throughout the region. (C) *NPF*>*GFP* expression colocalizes with the pan-enteroendocrine cell marker prospero (anti-Pros, red). Asterisks denote pairs of enteroendocrine cells. Note that only one cell in a pair expresses *NPF*>*GFP*. (D) All Pros^+^ enteroendocrine cells (blue) in the copper cell region are either *NPF*>*GFP* ^+^ (green) or anti-AstC^+^ (red). Asterisks denote pairs of enteroendocrine cells marked by propsero (blue). Most commonly, enteroendocrine pairs contain one *NPF*>*GFP* ^+^ cell and one anti-AstC^+^ cell. Scale bars: 200μm in A, 20μm in B.
(TIF)

Figure S3 **EGF ligand expression is induced in the gastric epithelium and surrounding muscle following infection.** (A-B) Gurken is not expressed in the adult midgut. (A) Anti-Grk expression (red) is not detected in the copper cell region under baseline conditions or (B) 24 hrs after infection. (C) The *spi*>*GFP* reporter (red) is expressed in *esg-lacZ* ^*+*^ progenitor cells (green) in the copper cell region following a 24 hr infection. (D) The *vn-lacZ* reporter (red) is induced in the visceral muscle (24B>*GFP* ^+^, green) surrounding the gastric epithelium following a 24 hr infection. (E) Quantification of *vn>lacZ* ^*+*^ cells pixel intensities in both the copper cell region and posterior midgut under baseline and infected conditions. A significant increase in expression is observed in both the copper cell region and posterior midgut following *Pe* infection (n=60 cells/condition, p<0.0001). (F) Anti-dpERK is induced in GSSCs that express the constitutively active form of the Raf kinase (arrows). Scale bar: 20μm in A and F.
(TIF)

Figure S4 **EGF is sufficient to promote gastric stem cell proliferation.** (A) The conditional *esg* ^*ts*^ driver line was used to express either *GFP* or *Raf* ^GOF^ . The number of *esg* ^+^, pH3^+^ cells in the copper cell region (CCR) was assayed 1-6 days after shifting to the restrictive temperature (n=15-16 guts/genotype/day, error bars ±SEM). (B) An increase in gastric stem cell proliferation is only observed when Gal4 and EGF-activating UAS constructs are expressed in the same fly. Baseline levels of proliferation are observed in flies that express the driver line alone (*esg^Gal4^*) or in flies only carrying a UAS construct (*UAS-Egfr^Act^, UAS-Ras^V12^, UAS-Raf^GOF^*). A significant increase in proliferation over baseline is only observed when the driver line and a UAS construct are expressed in the same animal (*esg* ^*ts*^ *>Egfr* ^*Act*^ *, esg* ^*ts*^>*Ras* ^V12^ *, esg* ^*ts*^>*Raf* ^*GOF*^; n=14-26 guts/genotype, error bars ±SEM).
(C) The MARCM system was used to label adult cell lineages in the copper cell region. The number of cells per clone was analyzed 14 days after induction (n=72-92 clones/genotype). There was no significant difference in the number of cells/clone when EGF signaling was activated in gastric stem cell lineages. (D) Distribution of clone size. *Egfr* ^*Act*^ and *Raf* ^GOF^ gastric stem cell lineages produced a larger percentage of big clones compared to controls (15-20 cells/clone for *Egfr* ^*Act*^; 20+ cells/clone for *Raf* ^*GOF*^).
(TIF)

Figure S5 **EGF signaling is not required for gastric stem cell fate specification.** (A-I) Fully differentiated copper and interstitial cells are anti-Dve^+^ and have large polyploid nuclei. Fully differentiated enteroendocrine cells are diploid and express prospero. (A, G) Control lineages in the adult copper cell region contain anti-Dve^+^ and anti-Pros^+^ cells. (B-F, H-I) Loss of EGF function in GSSC lineages does not affect anti-Dve and anti-Pros expression. Scale bar: 20μm.
(TIF)

Figure S6 **Notch/Delta signaling is induced following infection.** Flies carrying the reporter lines *Dl>GFP* or *Su*(H)*GBE*>*GFP* were infected with *Pe* for 24 hrs to induce proliferation. The number of dividing cells (pH3^+^) was scored with respect to GFP expression. The majority of pH3^+^ cells in the copper cell region are *Dl*>*GFP* ^+^ following challenge.
(TIF)
