# Supplementary figures and images for: Regional Control of Drosophila Gut Stem Cell Proliferation: EGF Establishes GSSC Proliferative Set Point & Controls Emergence from Quiescence
Source: PLoS One. 2013 Nov 13;8(11):e80608. doi: 10.1371/journal.pone.0080608 (PMC3827418; doi:10.1371/journal.pone.0080608)

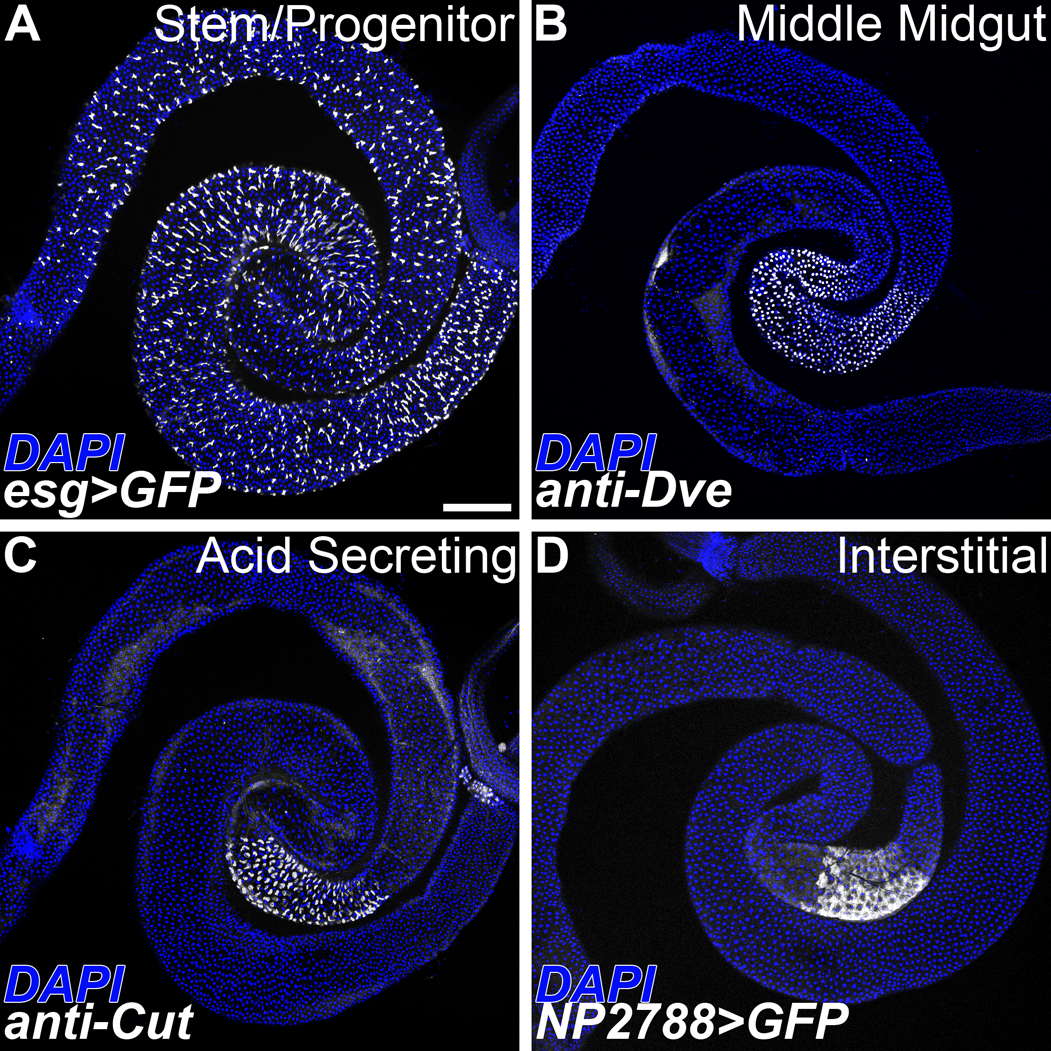

Supplement: Figure S1 — Molecular markers define specific cell types in the adult Drosophila copper cell region. (A) esg>GFP marks diploid progenitor cells throughout the entire length of the adult midgut. (B) Anti-Dve expression defines the middle midgut region and marks all polyploid differentiated cells in the region. (C) Anti-Cut marks the acid-secreting copper cells in a subdomain of the middle midgut. (D) NP2788>GFP is expressed in a subset of interstitial cells found in the middle midgut. Anterior is to the left in all panels. Scale bar: 200μm. (TIF) [file pone.0080608.s001.tif]

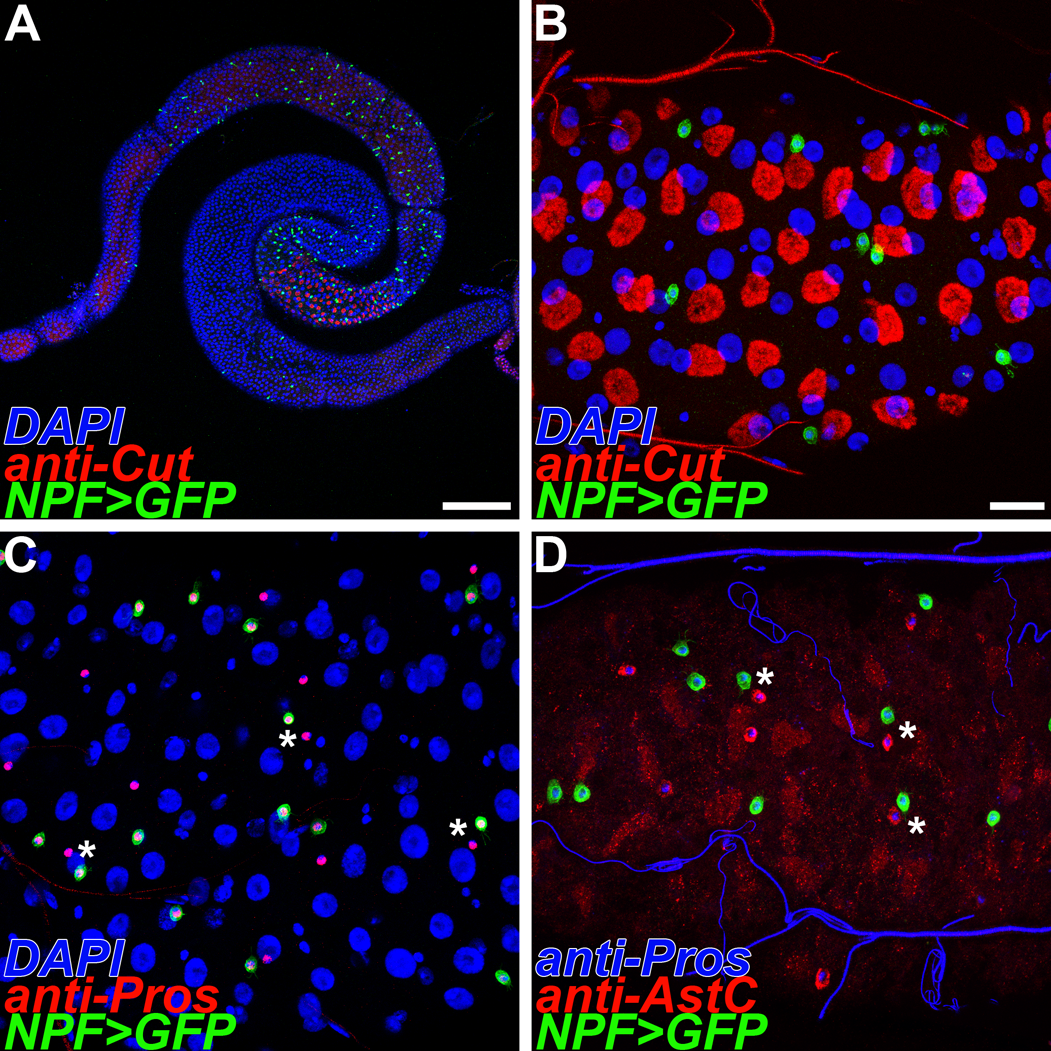

Supplement: Figure S2 — Enteroendocrine cells in the copper cell region can be further characterized based on neuropeptide expression. (A) The neuropeptide F (NPF) Gal4 driver (NPF>GFP +, green) is expressed in the copper cell region and in the flanking anterior and posterior midgut regions. Anti-Cut staining (red) marks the copper cell region of the middle midgut. Anterior is to the left. (B) High magnification image of the copper cell region (copper cells are marked by anti-Cut, red). The NPF diver is expressed in diploid cells throughout the region. (C) NPF>GFP expression colocalizes with the pan-enteroendocrine cell marker prospero (anti-Pros, red). Asterisks denote pairs of enteroendocrine cells. Note that only one cell in a pair expresses NPF>GFP. (D) All Pros+ enteroendocrine cells (blue) in the copper cell region are either NPF>GFP + (green) or anti-AstC+ (red). Asterisks denote pairs of enteroendocrine cells marked by propsero (blue). Most commonly, enteroendocrine pairs contain one NPF>GFP + cell and one anti-AstC+ cell. Scale bars: 200μm in A, 20μm in B. (TIF) [file pone.0080608.s002.tif]

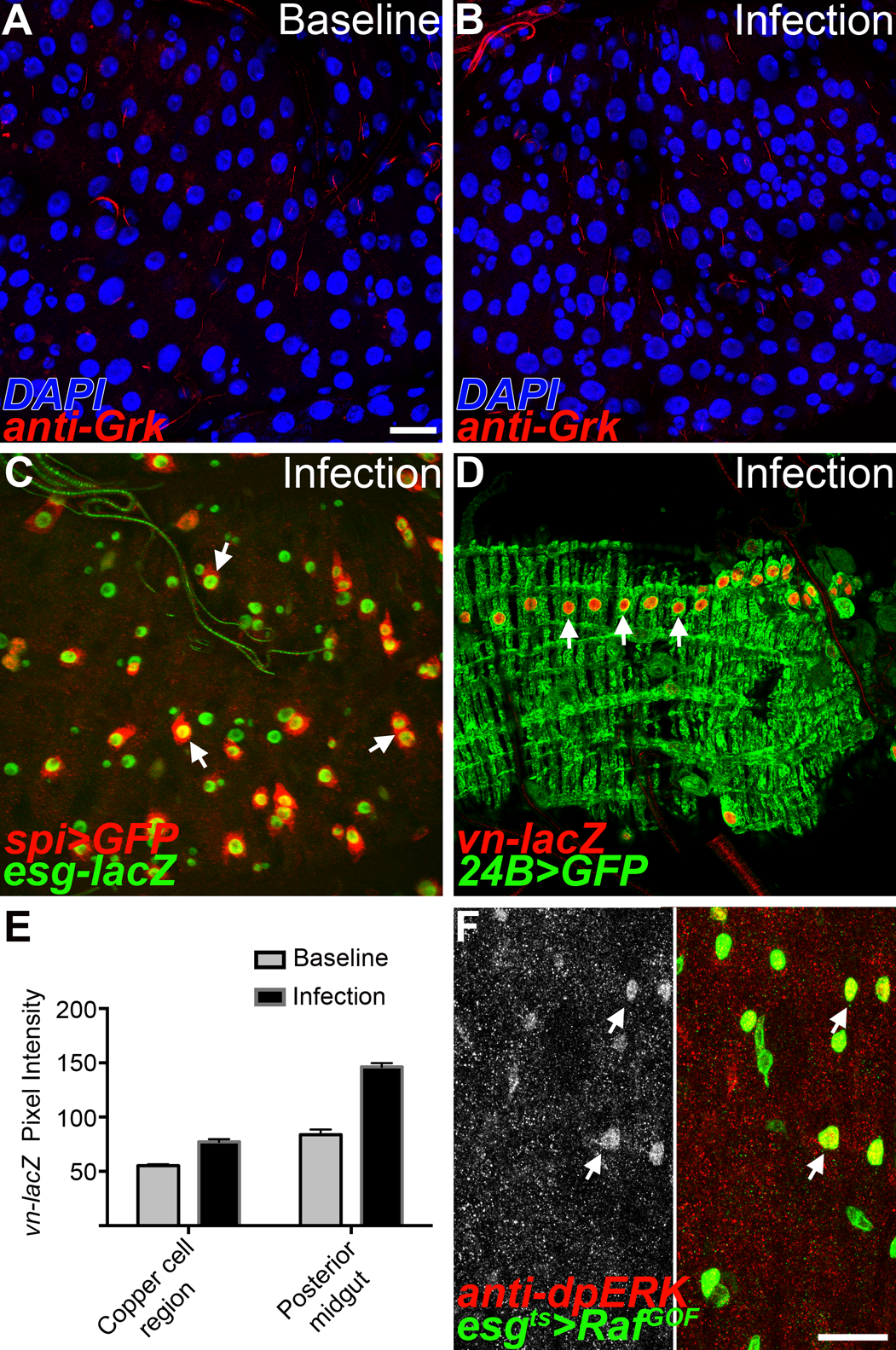

Supplement: Figure S3 — EGF ligand expression is induced in the gastric epithelium and surrounding muscle following infection. (A-B) Gurken is not expressed in the adult midgut. (A) Anti-Grk expression (red) is not detected in the copper cell region under baseline conditions or (B) 24 hrs after infection. (C) The spi>GFP reporter (red) is expressed in esg-lacZ + progenitor cells (green) in the copper cell region following a 24 hr infection. (D) The vn-lacZ reporter (red) is induced in the visceral muscle (24B>GFP +, green) surrounding the gastric epithelium following a 24 hr infection. (E) Quantification of vn>lacZ + cells pixel intensities in both the copper cell region and posterior midgut under baseline and infected conditions. A significant increase in expression is observed in both the copper cell region and posterior midgut following Pe infection (n=60 cells/condition, p<0.0001). (F) Anti-dpERK is induced in GSSCs that express the constitutively active form of the Raf kinase (arrows). Scale bar: 20μm in A and F. (TIF) [file pone.0080608.s003.tif]

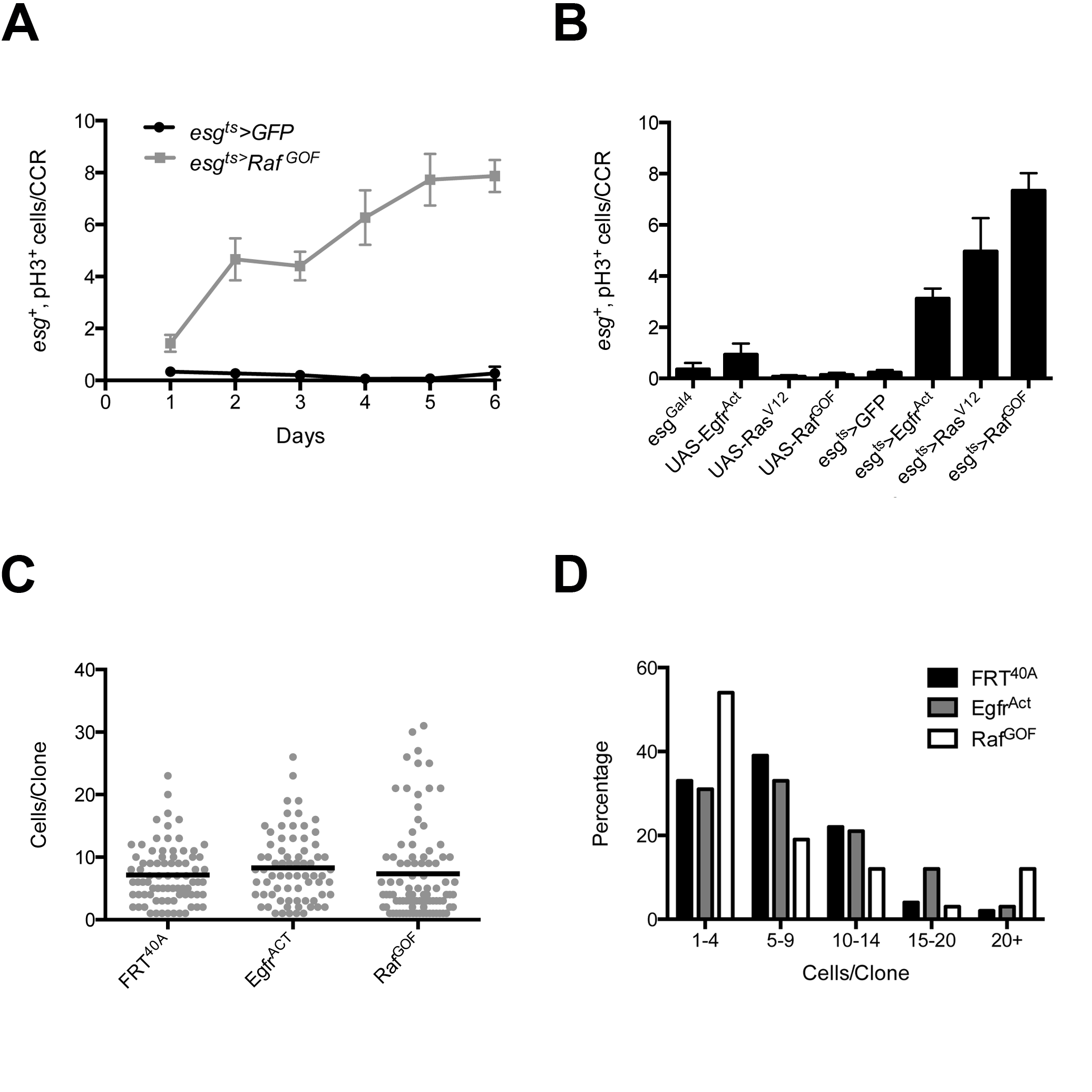

Supplement: Figure S4 — EGF is sufficient to promote gastric stem cell proliferation. (A) The conditional esg ts driver line was used to express either GFP or Raf GOF . The number of esg +, pH3+ cells in the copper cell region (CCR) was assayed 1-6 days after shifting to the restrictive temperature (n=15-16 guts/genotype/day, error bars ±SEM). (B) An increase in gastric stem cell proliferation is only observed when Gal4 and EGF-activating UAS constructs are expressed in the same fly. Baseline levels of proliferation are observed in flies that express the driver line alone (esgGal4) or in flies only carrying a UAS construct (UAS-EgfrAct, UAS-RasV12, UAS-RafGOF). A significant increase in proliferation over baseline is only observed when the driver line and a UAS construct are expressed in the same animal (esg ts >Egfr Act , esg ts>Ras V12 , esg ts>Raf GOF; n=14-26 guts/genotype, error bars ±SEM). (C) The MARCM system was used to label adult cell lineages in the copper cell region. The number of cells per clone was analyzed 14 days after induction (n=72-92 clones/genotype). There was no significant difference in the number of cells/clone when EGF signaling was activated in gastric stem cell lineages. (D) Distribution of clone size. Egfr Act and Raf GOF gastric stem cell lineages produced a larger percentage of big clones compared to controls (15-20 cells/clone for Egfr Act; 20+ cells/clone for Raf GOF). (TIF) [file pone.0080608.s004.tif]

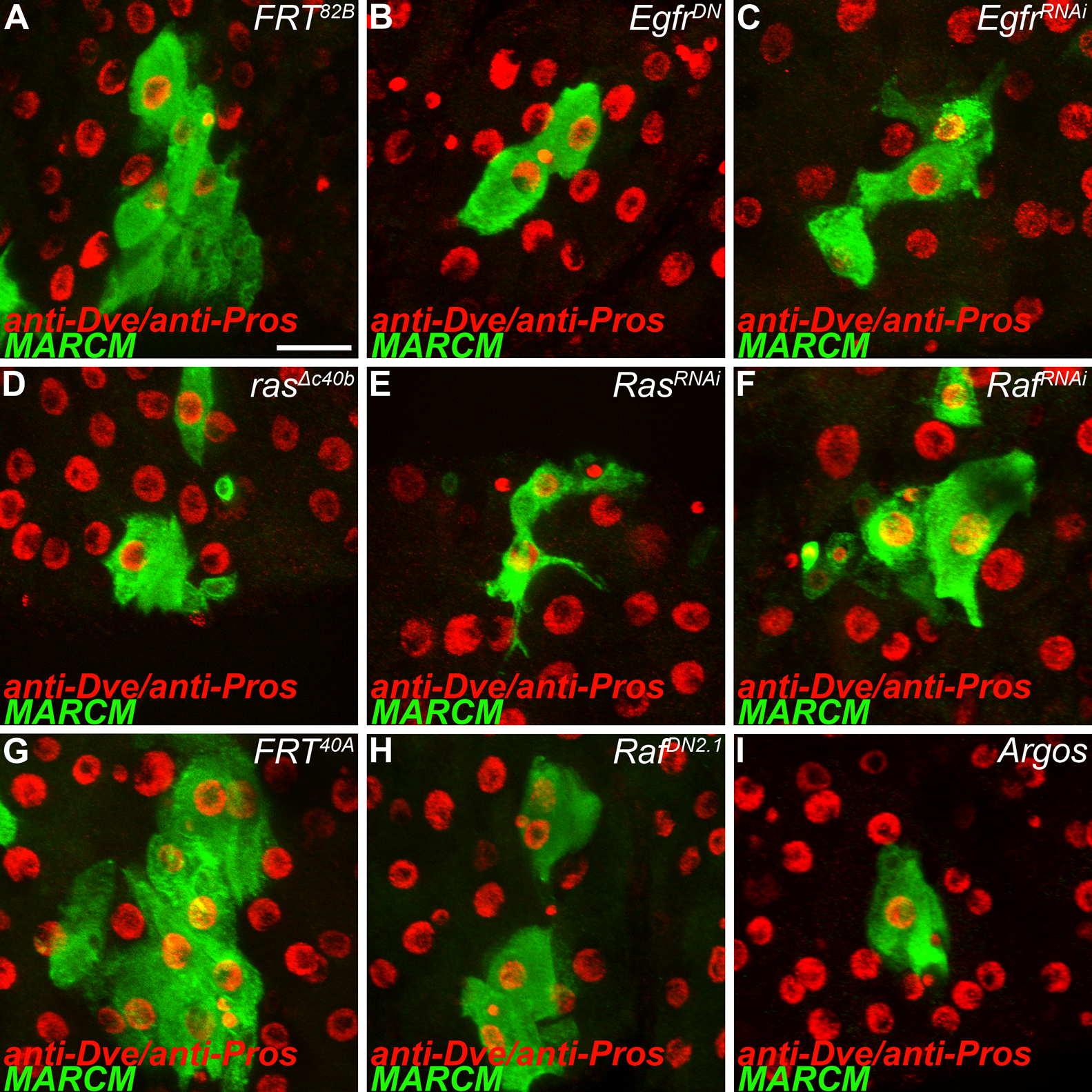

Supplement: Figure S5 — EGF signaling is not required for gastric stem cell fate specification. (A-I) Fully differentiated copper and interstitial cells are anti-Dve+ and have large polyploid nuclei. Fully differentiated enteroendocrine cells are diploid and express prospero. (A, G) Control lineages in the adult copper cell region contain anti-Dve+ and anti-Pros+ cells. (B-F, H-I) Loss of EGF function in GSSC lineages does not affect anti-Dve and anti-Pros expression. Scale bar: 20μm. (TIF) [file pone.0080608.s005.tif]

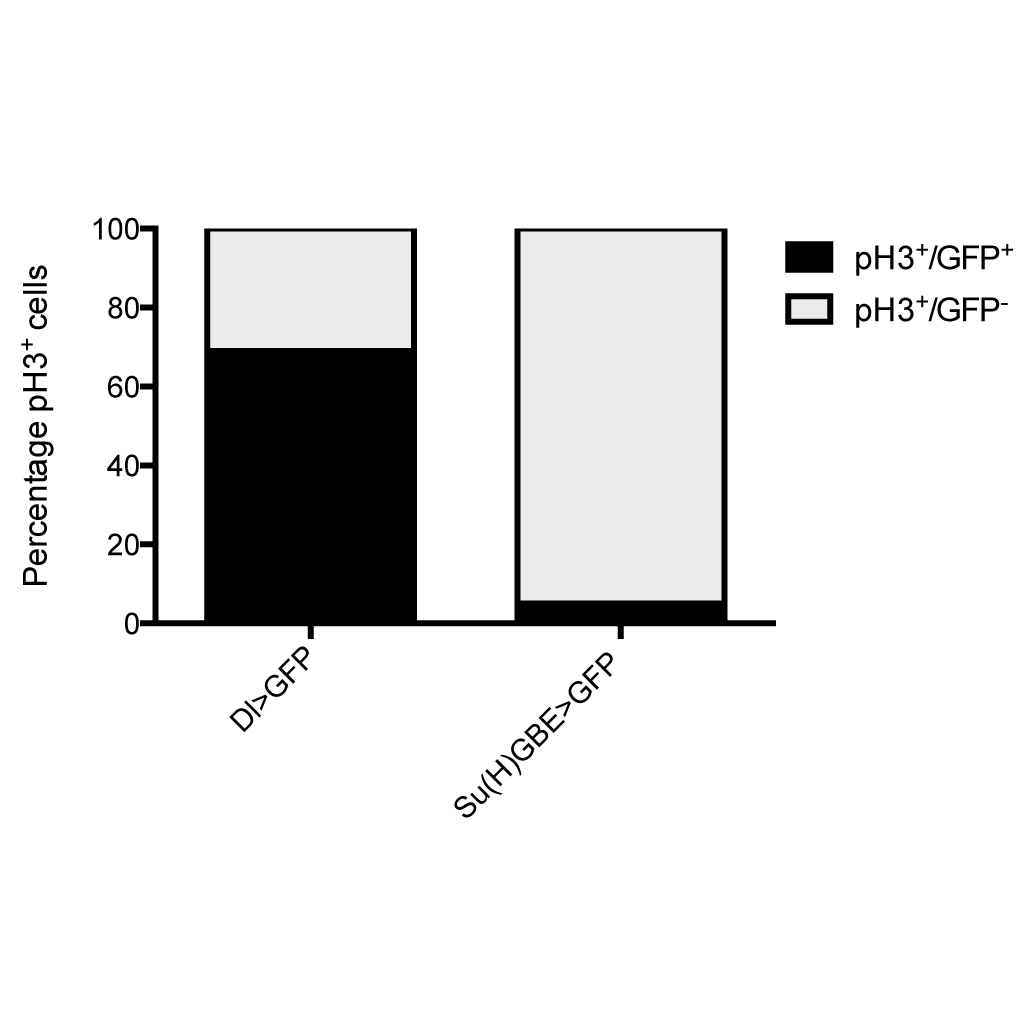

Supplement: Figure S6 — Notch/Delta signaling is induced following infection. Flies carrying the reporter lines Dl>GFP or Su(H)GBE>GFP were infected with Pe for 24 hrs to induce proliferation. The number of dividing cells (pH3+) was scored with respect to GFP expression. The majority of pH3+ cells in the copper cell region are Dl>GFP + following challenge. (TIF) [file pone.0080608.s006.tif]
